# Person-centred care to prevent hospitalisation - a focus group study with primary care providers

**DOI:** 10.1093/eurpub/ckac131.003

**Published:** 2022-10-25

**Authors:** C Nørby Lyhne, M Bjerrum, M Johansson Jørgensen

**Affiliations:** 1 Department of Public Health, Aarhus University, Aarhus C, Denmark; 2 Research Unit, Horsens Regional Hospital, Horsens, Denmark; 3 Center for Clinical Guidelines, Aalborg University, Aalborg, Denmark

## Abstract

**Background:**

The primary healthcare sector comprises various health services, including disease prevention at local level. Research shows that targeted primary healthcare services can prevent the development of acute complications and reduce the risk of hospitalisations. While interdisciplinary collaboration has been suggested as a means to improve the quality and responsiveness of personal care needs in preventive services, effective implementation remains a challenge. The aim was to investigate perceptions of preventive care among primary healthcare providers by examining their views on what constitutes a need for hospitalisation, and which strategies are found useful to prevent hospitalisation. Further, to explain how interdisciplinary collaboration can be supported with a view to providing person-centred care.

**Methods:**

Five focus group interviews were conducted with 27 primary healthcare providers. Interviews were transcribed, and analysed with qualitative content analysis.

**Results:**

Three categories emerged: 1) Mental and social conditions influence physical functioning and hospitalisation need, 2) Well-established primary healthcare services are important to provide person-centred care through interdisciplinary collaboration and 3) Interdisciplinary collaboration in primary healthcare services is predominantly focussed on handling acute physical conditions.

**Conclusions:**

By focusing on the whole person, it could be possible to provide more person-centred care through interdisciplinary collaboration and ultimately prevent some hospitalisations. The findings have clear implications for person-centred care and health system quality, and they highlight the need for involving stakeholders at all levels and for informing about the relevance of social and mental conditions, as they may influence the general health state and the risk of hospitalisation. The study supplements existing knowledge by providing valuable insight into the views of key primary healthcare providers.

**Key messages:**

• Healthcare providers are attentive towards the influence of mental, social and physical conditions on the risk of hospitalisation highlighting the importance of care that considers the whole person.

• The development and sustainable implementation of person-centred care in local primary care settings may be supported by evidence-based practices and co-production trajectories.

